# *Petrocodon luteoflorus* (Gesneriaceae), a new species from karst region in Guizhou, China

**DOI:** 10.3897/phytokeys.157.32316

**Published:** 2020-08-26

**Authors:** Zhi-Wei Fan, Lei Cai, Jia-Wen Yang, Sheng-Hu Tang, Fang Wen

**Affiliations:** 1 Guizhou Botanical Garden, Guiyang CN-550004, Guizhou, China; 2 Guangxi Key Laboratory of Plant Conservation and Restoration Ecology in Karst Terrain, Guangxi Institute of Botany, Guangxi Zhuang Autonomous Region and Chinese Academy of Sciences, Guilin, CN-541006, Guangxi, China; 3 Yunnan Key Laboratory for Integrative Conservation of Plant Species with Extremely Small Populations, Kunming Institute of Botany, Chinese Academy of Sciences, Kunming CN-650201, Yunnan, China; 4 University of Chinese Academy of Sciences, CN-CN-100049 Beijing, China; 5 Key Laboratory for Plant Diversity and Biogeography of East Asia, Kunming Institute of Botany, Chinese Academy of Sciences, Kunming CN-650201, Yunnan, China; 6 Gesneriad Conservation Center of China (GCCC), Guilin CN-541006, Guangxi, China

**Keywords:** Didymocarpoideae, limestone area, *
Petrocodon s. l.*, new taxon, flora of Guizhou

## Abstract

A new species of Gesneriaceae, *Petrocodon luteoflorus* Lei Cai & F.Wen was first described and illustrated from Maolan National Nature Reserve, Libo County, Guizhou Province, China. The diagnostic characters and notes of this species between its most morphologically similar species, *P. dealbatus* Hance, a detailed description, colour photographs, etymology, as well as distribution and habitat, are also provided in this paper.

## Introduction

Since *Petrocodon longistylus* Kraenzl., 1928 was merged into *P. dealbatus* Hance, 1883, *Petrocodons. str.* remained as a monotypic genus for over a century. However, two new species, *P. ferrugineus* Y.G. Wei, 2007 and *P. multiflorus* Fang Wen & Y.S. Jiang, 2011, were discovered and published after the beginning of the 21^st^ century. They have similar small white bell-shaped flowers. Thus, this genus became a small one and three species and one variety (P. dealbatus var. denticulatus (W.T. Wang) W.T. Wang) (Wang 1975, Wang et al. 1990, 1998, Wei et al. 2010) exist at that time. Soon afterwards, the genus was redefined based on molecular phylogenetic studies. The small Chinese genus *Petrocodon* has been recently enlarged to include four former monotypic genera (*Calcareoboea* C.Y. Wu ex H.W. Li, *Dolicholoma* D. Fang & W.T. Wang, *Paralagarosolen* Y.G. Wei & *Tengia* Chun), all species of *Lagarosolen* W.T. Wang, a few species of *Didymocarpus* Wall., one species of *Wentsaiboea* D. Fang & D.H. Qin (Weber et al. 2011) and one species of *Primulina* Hance (Xu et al. 2014). Thus, *Petrocodons.l.* consists of at least 35 species and one variety, including 14 species newly described after 2011 (Lu et al. 2017a, Xu et al. 2017), e.g. *P. asterocalyx* F. Wen, Y.G. Wei & R.L. Zhang, 2018, *P. pulchriflorus* Y.B. Lu & Q. Zhang, 2017a, *P. urceolatus* F.Wen, H.F. Cen & L.F. Fu, 2017, *P. retroflexus* Q. Zhang & J. Guo, 2016, and so on. Obviously, the genus is so special on account of its remarkable and highly variable floral structures that it also becomes one of the most taxonomically difficult groups in Gesneriaceae (Möller et al. 2016, Lu et al. 2017b). For example, *P. guangxiensis* (Yan Liu & W.B. Xu) W.B. Xu & K.F. Chung was mistakenly identified as a member of *Primulina* Hance, 1883, *P. guangxiensis* Yan Liu & W. B. Xu (Liu et al. 2011, Xu et al. 2014) while it was published. On the other hand, similar characters of leaves sometimes affect our judgement of some *Petrocodon* congeners.

During field investigations in the karst region of Guizhou province in 2017, an interesting species of Gesneriaceae attracted our attention. Previously, the corresponding author (FW) also collected specimens of the same species without flowers from Jiudongtian, Dongtang town, Libo County, Guizhou. After we checked the flowering plants which were being cultivated in Guilin Botanical Garden and Guizhou Botanical Garden, we confirmed that it is a member of *Petrocodon* because the morphology of plants and flowers is similar to *P. dealbatus*. Subsequently, we re-collected the flowering specimens from Maolan National Nature Reserve, Libo County, Guizhou province in 2018. After careful review of the relevant specimens and literature of *Petrocodon*, we concluded that this unknown species represents a species new to botany and science which we describe and illustrate here and its morphological characters are compared with the closely related species *P. dealbatus*.

## Material and methods

All available specimens of *Petrocodons.l.* stored in the herbaria (IBK, KUN and PE) in China were examined. The photographs were taken in the field by the authors. Morphological observations, measurements and description of the new species were carried out based on living plants, dry specimens and preserved materials. All morphological characters were studied with dissecting microscopes and are described using the terminology presented by Wang et al. (1990, 1998), Li and Wang (2004).

## Taxonomy treatments

### Petrocodon luteoflorus

Taxon classificationPlantaeLamialesGesneriaceae

Lei Cai & F.Wen sp. nov.

30B0C950-88E0-59D6-A8F3-9F8D714A68AD

urn:lsid:ipni.org:names:77211193-1

Fig. 1

#### Diagnosis.

*Petrocodon luteoflorus* most resembles *P. dealbatus* in plant type and floral size, but can be easily distinguished from the latter by the following diagnostic characters: longer calyx lobes 6–8 mm long (vs. 2–5 mm long); larger corolla 9–11 mm long, pale yellow to yellow (vs. 5.5–8 mm long, white); corolla lobes in equal shape and size, triangular and ca. 2 mm long (vs. in different shape and size: adaxial lobes 0.8–2 mm long, triangular; abaxial lobes 1.8–3 mm long, triangular to ovate).

**Figure 1.**
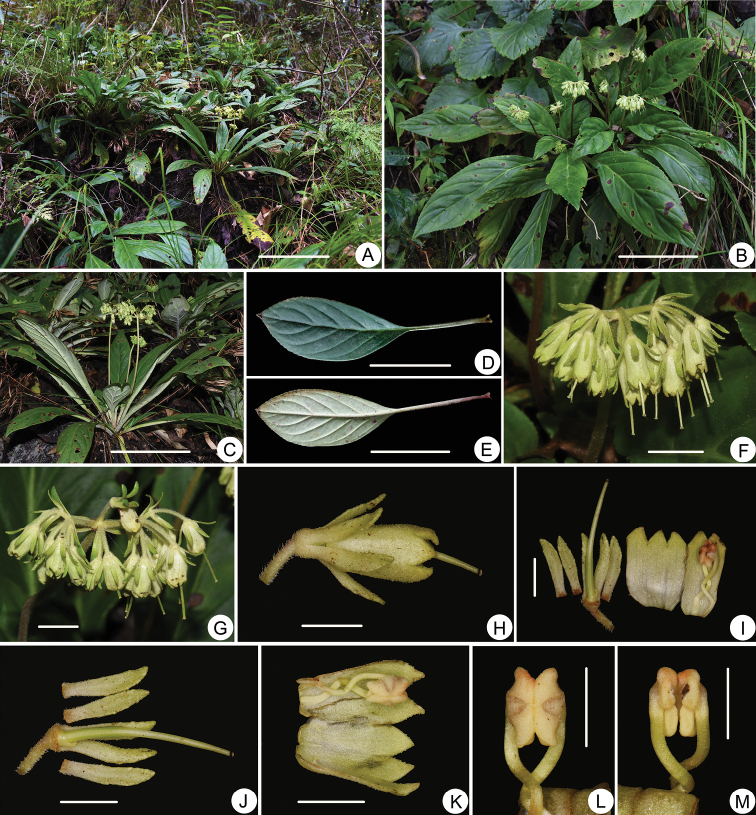
*Petrocodon luteoflorus* Lei Cai & F.Wen, sp. nov. **A** Habitat **B, C** plants with flowers **D** petiole and adaxial leaf surface **E** petiole and abaxial leaf surface **F, G** gymes **H** side view of flower **I** opened corolla and pistil with calyx **J** pistil, disc and calyx **K** opened corolla showing stamens and staminodes **L** adnate anthers, adaxial view **M** adnate anthers, abaxial view. Scale bars: 20 cm (**A**); 10 cm (**B, C**); 5 cm (**D, E**); 1 cm (**F, G**); 5 mm (**H, I, J, K**), 3 mm (**L, M**). Photographed by Lei Cai and Fang Wen (**D, E**).

#### Type.

CHINA. Guizhou: Libo County, Limingguan Town, Yaolan, 25°17'N, 107°55'E, 735 m a.s.l., on moist rocks under forest, in flower, 23 August 2018, *Lei Cai* et al. *CL193* (holotype: KUN!, isotypes: KUN! & IBK!).

#### Description.

Perennial herb, stemless. Rhizome 8–12 cm long, 5–12 mm in diameter. Leaves 8–16, basal; petiole 3.5–6.5 cm long, densely pruinate; leaf blade narrow elliptic or oblanceolate, 8–20 × 2.5–8 cm, coriaceous, adaxially green, appressed pubescent, abaxially appressed pubescent along veins, whitish-green, pruinate, base cuneate, gradually tapered to petiole, margin nearly entire to denticulate or crenulate, apex acuminate; lateral veins 4–6 on each side of midrib. Cymes axillary, 1–5 on a plant, flowers numerous, 8–25-flowered or more; peduncle 8–20 cm long, densely puberulent; bracts 2, lanceolate, 8–10 × 1.5–2 mm, both sides puberulent, margin entire, apex obtuse. Pedicel 0.8–2.2 cm long, pubescent. Calyx 6–8 mm long, 5-lobed to the base; lobes equal, linear, 6–8 × 1.5–2.0 mm, outside pubescent, inside glabrous, margin entire to denticulate, apex acuminate. Corolla slightly or inconspicuously 2-lipped, pale yellow to yellow, 9–11 mm long, 4–6 mm in diameter, outside densely puberulent, inside glabrous; tube cannulate, 7–9 × 4–6 mm; adaxial lobes 2, abaxial lobes 3, all lobes triangular, in nearly equal size, ca. 2 mm long, 2.5 mm wide at the base. Filaments ca. 7 mm long, S-shaped, glabrous, inserted ca. 2 mm from base; anthers dorsifixed, ca. 3 mm long, reniform or water-chestnut shaped, apexes acute; staminodes 3, ca. 0.5 mm long, linear, glabrous, inserted ca. 1 mm from base. Disc ca. 1.5 mm high, margin asymmetrical, one side absent, on the other side horned. Pistil ca. 1.5 cm long, glabrous; ovary linear, ca. 6 mm long, style linear, ca. 9 mm long; Stigma discoid, small, 0.3–0.5 mm in diameter. Capsule linear, glabrous, 2–3 cm long.

#### Phenology.

Flowering from August to September; fruiting from September to November.

#### Etymology.

The specific epithet ‘*luteoflorus*’ derives from the Latin prefix, *luteo*-, yellow and the Latin suffix, ‘-*florus*’, of flower, referring to its small and yellow flowers of the new species. The Chinese name is “Xiǎo Huáng Huā Shí Shān Jù Tái” (小黄花石山苣苔).

#### Distribution and habitat.

*Petrocodon luteoflorus* is currently known only from the type locality and might be endangered but more data are needed to evaluate that reliably. The species only grows on the surface of moist rocks under the forest.

#### Conservation status.

Current information for this new species is known from only a few collections and details on the size of the population are known in Maolan National Nature Reserve, where the plants’ protected status is guaranteed. Based on some careful field investigations in recent years, this species appears to be locally abundant. Thus, it is assessed temporarily as Least Concern (LC) according to the IUCN Red List Categories and Criteria (IUCN 2017).

#### Additional specimens examined.

Guizhou: Libo County, Dongtang Town, Raosuo village, Jiudongtian, 25°17'N, 103°03'E, 795 m a.s.l., on moist rocks at the entrance of limestone caves, 11 September 2017, *Fang Wen WF160113-01* (IBK!).

#### Notes.

**Figure 2.**
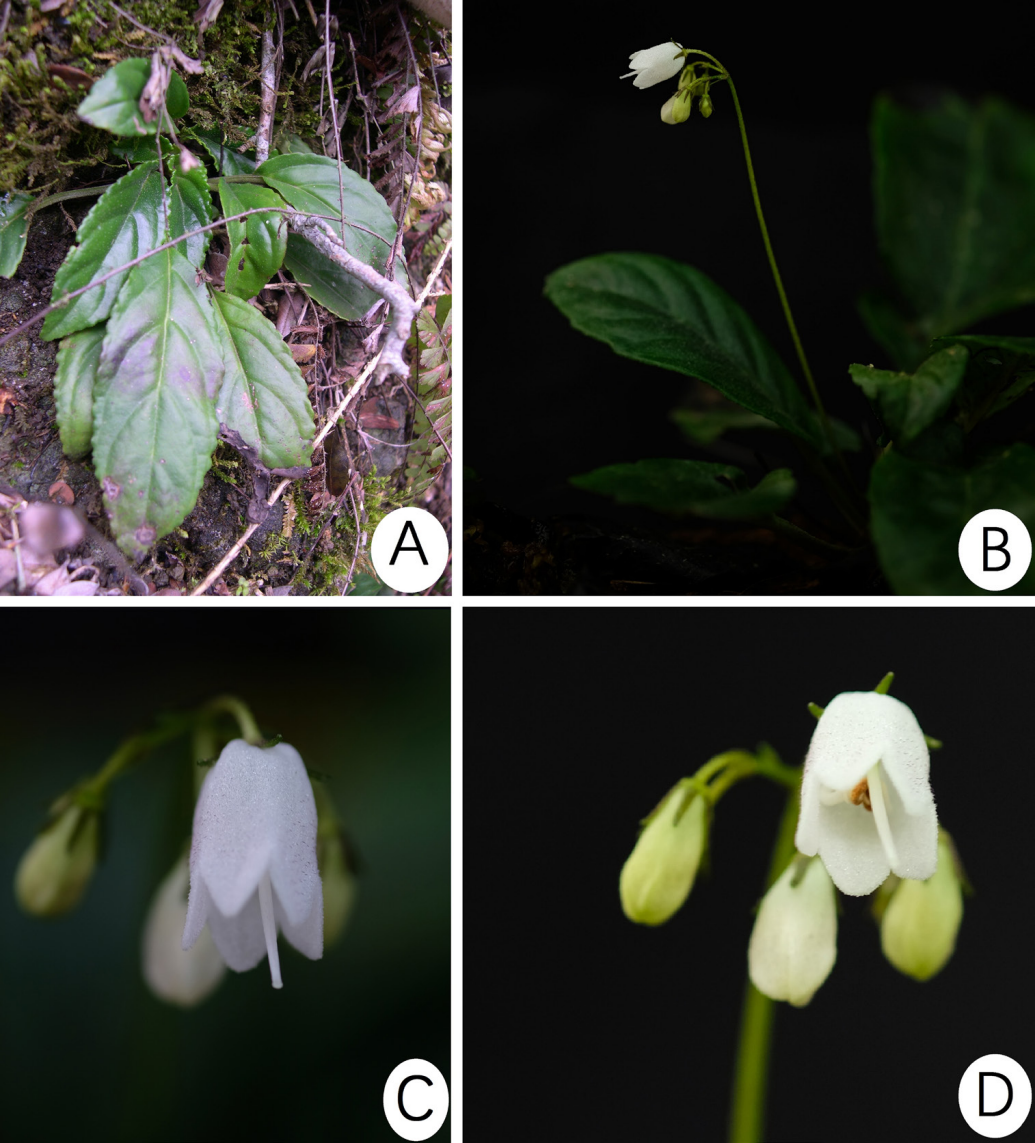
*Petrocodon dealbatus* Hance. **A** Habitat **B** cyme **C** the lateral view corolla and calyx lobes **D** the frontal view of corolla. Photographed by Fang Wen.

At first glance, this new taxon and the type species, *Petrocodon dealbatus* (Fig. 2), could easily have been confused because of its similar leaves if the pruinate abaxial leaf surfaces escaped collectors’ attention or were not in flower. However, once the flowers of the two congeners are compared to each other, they are easy to distinguish. The new species can be easily distinguished from the latter in the shape and length of bracts (lanceolate, 8–10 mm long in *P. luteoflorus* vs. linear, 3–7 mm long in *P. dealbatus*); the length of calyx lobes (6–8 mm long vs. 2–5 mm long); the size and colour of corolla (9–11 mm long, pale yellow to yellow vs. 5.5–8 mm long, white); the shape and size of corolla lobes in equal shape and size: triangular and ca. 2 mm long vs. in different shape and size: adaxial lobes 0.8–2 mm long, triangular; abaxial lobes 1.8–3 mm long, triangular to ovate); indumentum of corolla outside (densely puberulent vs. upper half of corolla puberulent to glabrescent); filaments (S-shaped vs. straight); shape of anthers (reniform or water-chestnut shaped vs. elliptic); the shape and height of disc (ca. 1.5 mm high, asymmetrical, one side absent, on the other side horned vs. ca. 1 mm high, symmetrical and annular).

## Supplementary Material

XML Treatment for Petrocodon luteoflorus
